# Capsule Endoscopy in Celiac Disease

**DOI:** 10.1155/2012/676073

**Published:** 2011-12-22

**Authors:** E. Akin, O. Ersoy

**Affiliations:** Department of Gastroenterology, Ankara Ataturk Research and Education Hospital, 06800 Ankara, Turkey

## Abstract

Capsule endoscopy (CE) has been increasingly used for diagnosing disease of the small bowel. It is an attractive technique for assessing celiac disease (CD) because it is noninvasive and provides a close and magnified view of the mucosa of the entire small bowel. The aim of this paper is to update the current data on the use of CE for diagnosing villous atrophy and complications of CD.

Celiac disease (CD) is a chronic autoimmune enteropathy occurring in genetically predisposed individuals following ingestion of wheat gluten and related protein fractions of other grains [1]. CD is the most frequently seen enteropathy in western countries, and its prevalence is 0.7–2% [2]. Patients present with diarrhea, weight loss, steatorrhea, or malnutrition syndromes such as anemia and diminished bone mass due to deficiencies of important nutrients (iron, folate, calcium, and fat-soluble vitamins) [3]. However, the increased interest for this pathology over the last 2 decades allowed diagnosing CD also in those with the silent or “atypical” form. These patients may present vague and subclinical manifestations such as dyspeptic symptoms or esophageal reflux, irritable bowel syndrome, polyneuropathy, or iron deficiency anemia [4]. The analogy of an iceberg was suggested for CD, meaning that only a small portion of patients with classic symptoms are diagnosed, whereas the majority of asymptomatic individuals or subjects with mild, nonspecific symptoms remain undiagnosed and untreated [5]. On the other hand, growing body of evidence shows that early diagnosis and treatment can reduce the risk of malignant complications, such as lymphoma [6].

The first step in pursuing a diagnosis for CD is a serological test. Serologic tests, particularly the immunoglobulin A (IgA) antiendomysial (AEA) and the IgA tTGA, have become a relatively sensitive and specific way to initially detect CD. Many studies demonstrate a specificity of IgA tTGA greater than 95% and a sensitivity in the range of 90%–96%. AEA has a slightly lower and variable sensitivity but an excellent specificity (99,6%). Although the sensitivity and specificity of these tests are high, false-negative results can occur in mild enteropathy and in patients with IgA deficiency [7]. By contrast, antigliadin antibody (AGA) tests are no longer used routinely because of their lower sensitivity and specificity. However, a second generation AGA test (Deamidated Gliadin Peptide (DGP)) yielded far higher diagnostic accuracy (sensitivity 94 percent, specificity 99 percent) [8].

 Genetic testing may be helpful for the diagnosis. It is well known that CD is strongly associated with specific HLA class II genes known as HLA-DQ2 and HLA DQ8 located on chromosome 6p21. Most CD patients (around 90%) express HLA-DQ2 and the remaining patients are usually HLA DQ8 positive. However, it is well known that only around 3–5% of DQ2- or DQ8-expressing patients actually develop CD. Thus, HLA DQ2 or HLA DQ8 is necessary for disease development but not sufficient. Non-HLA genes contribute more than HLA to the CD genetic background. However, this predisposition depends on a multitude of genes, each of them adding only a modest contribution to disease development. Several genome-wide studies have implicated strong candidate regions for alternative susceptibility loci including 11p11, 5q31, and 19q13.4 [9].

The gold standard for the diagnosis of CD is histopathology of the small bowel. A small intestinal biopsy, which typically shows villous atrophy, increased intraepithelial lymphocytes and hyperplastic crypts in patients with CD [10]. Endoscopic markers suggestive of CD are reduction in number or loss of Kerckring's folds, mosaic pattern, scalloped folds, and visibility of the underlying blood vessels. These signs cannot reliably predict CD. The reported specificity for endoscopic markers ranges from 87% to 100% but this markers are considered to lack sensitivity with a reported range from 50% to 94% [11, 12]. Recognition of endoscopic signs of CD could help to select patients for biopsy and avoid delays in the diagnosis of the disease, preventing long-term complication.

Duodenal biopsy may be limited by patient's aversion to undergo upper gastrointestinal (GI) endoscopy, especially in asymptomatic patients; other limitations include the difficulty of obtaining adequate and properly oriented tissue samples, the occurrence of patchy mucosal lesions that can be missed by the biopsy, and, in some cases, the most severe mucosal changes occur in the jejunum, which is not accessible to conventional upper GI endoscopy [13].

Capsule endoscopy (CE) is a diagnostic imaging method used in several intestinal diseases [14–22]. It produces high-quality images of the small bowel mucosa, with an eightfold magnification and has been shown to be superior to other diagnostic tools for the diagnosis of a variety of diseases, including refractory CD [17, 23–30]. The main advantages of CE are that it is noninvasive, it images the entire length of the small bowel, and it is able to detect minute mucosal details, including changes in intestinal villi. 

There are frequently published studies on the role of CE in the diagnosis of CD. In general, most endoscopic markers of CD as described in literature are seen with greater clarity by CE. At CE, the mucosa in CD may appear scalloped. The mosaic appearance of the mucosa is also apparent. In CD, the villi may appear shortened and thickened, layered or stacked folds. In addition to general mucosal pattern, CE can easily recognize finger-like villi [22, 31]. We observed scalloping of the folds, mosaic pattern, nodularity, and layering of folds in CE of our patients with CD (Figure 1). 

Petroniene et al. showed that the extent of bowel involvement appears to correlate with the severity of symptoms [10]. Rondonotti et al. exploited the capability of the CE to evaluate the longitudinal extent of mucosal involvement beyond the duodenum and its correlation with clinical and biochemical parameters [32]. In fact, Murray and colleagues demonstrated that the length of bowel involved did not correlate with the mode of symptomatic presentation in patients [33].

A few studies have been published comparing duodenal biopsy and CE regarding the villous atrophy occurring in intestinal mucosa in CD (Table 1) [32, 34–38]. Petroniene et al. compared 10 CD patients with histologically proven villous atrophy with 10 control patients with normal histology. Sensitivity, specificity, positive predictive value (PPV), and negative predictive value (NPV) of CE in diagnosing villous atrophy were 70%, 100%, 100%, and 77%, respectively [34].

Hopper et al. showed that 17 out of 20 patients with CD had villous atrophy also were detected by CE. In this paper, the sensitivity, specificity, PPV, and NPV for CE in recognising villous atrophy were 85%, 100%, 100%, and 88.9%, respectively. Upper GI endoscopy detected endoscopic markers consistent with CD in 16 out of 20 CDs with a sensitivity, specificity, PPV, and NPV of 80%, 100%, 100%, and 85.7%, respectively. CE was more sensitive than conventional endoscopy in identifying endoscopic markers, but the difference observed did not achieve statistical significance [35]. Rondonotti et al. compared CE with duodenal biopsy in patients with signs and/or symptoms suggestive of CD and positive serology. CE was reported to have a sensitivity of 87.5% and specificity of 90.9%, with PPV, and NPV of 96.5% and 71.4%, respectively, and positive and negative likelihood ratios of 9.6 and 0.14, respectively [32].

Biagi et al. did not confirm this data. In this study, the authors evaluated whether there was a correlation between the degree of villous atrophy at the histology and CE results. CE findings regarding the degree of small bowel mucosal atrophy showed only a moderate agreement with the histologic pattern, with a high sensitivity (90.5%–95.2%) but a low specificity (63.6%). PPV was 100% and NPV ranged 77.8% and 87.5% [36]. Maiden et al. compared CE with histological specimens of proximal small bowel in patients with CD who had failed to respond to a gluten-free diet. CE was reported to be normal in ten (53%) cases, have mild-moderate changes in three (16%) cases, and have moderate-severe changes in six (31%) cases. Endoscopy demonstrated concordance with histological changes in 14 of the 18 patients with histology available (78% concordance). Compared with distal duodenal biopsy, CE showed sensitivity 67%, specificity 100%, PPV 100%, and NPV 60% [37].

Lidums et al. compared suspected CD patients (positive celiac serology and normal duodenal histology) and known CD patients (positive celiac serology and villous atrophy), with CE, whether these patients have any endoscopic markers of CD. In this paper, the sensitivity, specificity, PPV, and NPV for CE in recognising villous atrophy were 93%, 100%, 100%, and 89%, respectively [38].

In our study, 8 untreated patients who had AEA positive and duodenal biopsy results consistent with CD were evaluated. We have shown that CE provided no diagnostic contribution to CD when compared with duodenal biopsy [22]. 

CE also may be helpful in patients with CD with symptomatic relapse or refractory CD and in elderly patients with atypical symptoms or chronic iron deficiency anemia [39]. Culliford et al. showed that, among 47 patients with complicated CD, almost 50% had lesions detected by CE. One adenocarcinoma was identified; however, ulceration was common [40]. 

The complications of long-standing CD include lymphoma, ulcerative jejunitis, and adenocarcinoma. Consequently, those patients develop a recurrence of diarrhea, fever, abdominal pain, or evidence of GI bleeding. These complications are often not identifiable by conventional imaging modalities as they are located beyond the site reachable by traditional endoscopy. CE should be performed early when these symptoms occur [31]. CE has been reported to be able to demonstrate intussusceptions, ulcerative jejunoileitis, lymphoma, and adenocarcinoma in patients with CD [31, 41, 42]. It is unclear which group of patients who have CD should undergo surveillance by CE to detect these malignancies. The authors suggest that candidates include elderly patients recently with CD. In addition, individuals diagnosed in childhood, who are rediagnosed as adults, may be at increased risk of adenocarcinoma [31].

In conclusion, CE provides diagnostic images of the small bowel mucosa in patients with CD. At present, CE offers an alternative to duodenal biopsy in patients unable or unwilling to undergo conventional upper GI endoscopy. In addition, CE should be considered in elderly patients with a new diagnosis of CD or those patients with persistent or relapsing symptoms.

## Figures and Tables

**Figure 1:**
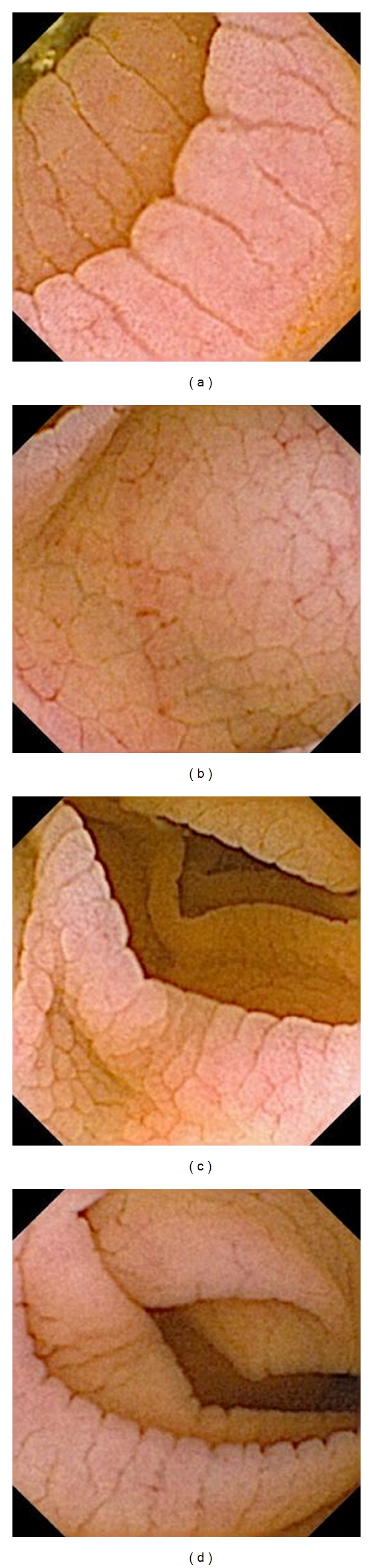
Capsule endoscopy images of celiac disease. (a) scalloping; (b) mosaic pattern; (c) micronodularity; (d) layering of folds.

**Table 1:** Summary of the trials sensitivity, specificity, PPV, NPV of capsule endoscopy in celiac disease.

Study	*n*	Country	Sensitivity	Specificity	PPV	NPV
Petroniene et al. [34]	10	Canada	70%	100%	100%	77%
Hopper et al. [35]	21	UK	85%	100%	100%	88.9%
Rondonotti et al. [32]	32	Italy	87.5%	90.9%	96.5%	71.4%
Biagi et al. [36]	26	Italy	90.5–95.2%	63.6%	100%	77.8–87.5%
Maiden et al. 37]	19	UK	67%	100%	100%	60%
Lidums et al. [38]	22	Australia	93%	100%	100%	89%

PPV: positive predictive value, NPV: negative predictive value.
